# miR-365 (microRNA): Potential Biomarker in Oral Squamous Cell Carcinoma Exosomes and Extracellular Vesicles

**DOI:** 10.3390/ijms21155317

**Published:** 2020-07-27

**Authors:** Jeffery Coon, Karl Kingsley, Katherine M. Howard

**Affiliations:** 1 Department of Clinical Sciences, Las Vegas—School of Dental Medicine, University of Nevada, 1001 Shadow Lane, Las Vegas, NV 89106, USA; coonj1@unlv.nevada.edu; 2 Department of Biomedical Sciences and Director of Student Research, Las Vegas—School of Dental Medicine, University of Nevada, 1001 Shadow Lane, Las Vegas, NV 89106, USA; 3 Department of Biomedical Sciences, Las Vegas—School of Dental Medicine, University of Nevada, 1001 Shadow Lane, Las Vegas, NV 89106, USA; katherine.howard@unlv.edu

**Keywords:** microRNA, miR-365, oral cancer, exosome, extracellular vesicle, liquid biopsy

## Abstract

Introduction: miR-365 is a non-coding microRNA that regulates transcription and has been demonstrated to promote oncogenesis and metastasis in some cancers, while suppressing these effects in others. Many microRNAs are produced and then exported extracellularly in exosomes, which are small extracellular vesicles ranging from 30 to 100 nm that are found in eukaryotic fluids and facilitate many cellular functions. Exosomes and extracellular vesicles are produced by many cell types, including oral cancer cells—although no study to date has evaluated miR-365 and oral cancer exosomes or extracellular vesicles. Based on this information, our research question was to evaluate whether oral cancers produce exosomes or extracellular vesicles containing miR-365. Materials and Methods: Two commercially available oral cancer cell lines (SCC25 and CAL27) and a normal oral keratinocyte (OKF4) were grown in serum-free media, supplemented with exosome-depleted fetal bovine serum. Extracellular vesicles and exosomes were then isolated using the Invitrogen total exosome RNA and protein isolation kit for processing using the hsa-miR-365a-5p microRNA qPCR assay kit. Results: RNA was successfully isolated from the exosome-depleted supernatant from each cell line—SCC9, SCC15, SCC25, and CAL27 (oral squamous cell carcinomas) and OKF4 (oral epithelial cell line). Relative concentrations of RNA were similar among each cell line, which were not significantly different, *p* = 0.233. RNA quality was established by A260:A280 absorbance using a NanoDrop, revealing purity ranging 1.73–1.86. Expression of miR-16 was used to confirm the presence of microRNA from the extracted exosomes and extracellular vesicles. The presence of miR-365 was then confirmed and normalized to miR-16 expression, which demonstrated an increased level of miR-365 in both CAL27 and SCC25. In addition, the normalized relative quantity (RQ) for miR-365 exhibited greater variation among SCC25 (1.382–4.363) than CAL27 cells (1.248–1.536). Conclusions: These results confirm that miR-365 is not only expressed in oral cancer cell lines, but also is subsequently exported into exosomes and extracellular vesicles derived from these cultures. These data may help to contextualize the potential for this microRNA to contribute to the phenotypes and behaviors of oral cancers that express this microRNA. Future research will begin to investigate these potential mechanisms and pathways and to determine if miR-365 may be useful as an oral cancer biomarker for salivary or liquid biopsies.

## 1. Introduction

miR-365 is a non-coding microRNA that regulates transcription and has been demonstrated to promote oncogenesis and metastasis in some cancers, while suppressing these effects in others [1,2,3]. For example, cancer cell growth, migration, and invasion were shown to be inhibited by miR-365 through interactions with multiple intracellular targets, including GALNT4 and ADAM10 [4,5]. In other examples, miR-365 may also function to promote the development of some cutaneous (skin) cancers by downregulating BAX and nuclear factor I/B (NFIB) [6,7,8].

Exosomes are small extracellular vesicles ranging from 30 to 100 nm found in eukaryotic fluids that facilitate many cellular functions, including modulation of tumor phenotypes within and between tumor cells [9,10]. Many microRNAs are produced and then exported extracellularly in exosomes [11,12]. Recent evidence now suggests that miR-365 may also function to modulate carcinogenesis and phenotypic behaviors in oral cancers through interactions with nuclear enriched abundant transcript 1 (NEAT1) [13,14,15]. In fact, preliminary studies from this group screened for the cytoplasmic expression of miR-365 among several oral cancer cell lines, some of which were previously tested (Cal27, Scc-9, and Scc-25) and others that had never been evaluated (Scc-4 and Scc-15), and found differential results [16] However, no studies to date have evaluated miR-365 expression among extracellular vesicles and exosomes derived from these specific cell lines. In addition, another related study from this group isolated exosomes and extracellular vesicles from oral cancer stem cells to screen for the expression of several microRNAs (miR-21, -31, -32, -34, -133, -155, and -365)—although only the expression of miR-21, -34, and -155 were observed among the exosomes specific to oral cancer stem cells [17].

Because the previous studies focused on cytoplasmic expression of miR-365 or were restricted to oral cancer stem cells, the current study sought to evaluate miR-365 expression within oral cancer exosomes and extracellular vesicles [18,19,20]. A greater understanding of the basic biology and role of exosomes and microRNAs in oral cancer is needed for both clinical cancer detection and treatment decisions. The elucidation and cataloguing of which microRNAs are produced and exported would advance this understanding [21,22,23]. Based on this information, our specific research question was to evaluate whether oral cancers produce exosomes or extracellular vesicles containing miR-365.

## 2. Results

**Figure 1:**
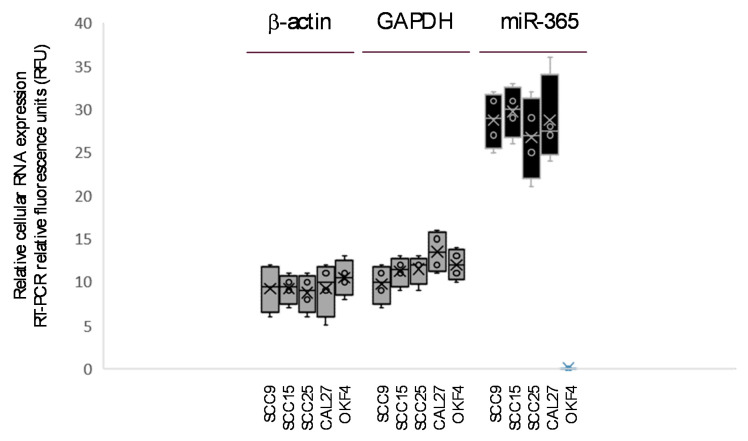
Screening of cellular RNA for miR-365. RT-PCR confirms the production of structural (β-actin) and metabolic (GAPDH) mRNA in all cells. Presence of miR-365 was restricted to oral cancer cell lines (SCC9, SCC15, SCC25, CAL27) at levels exceeding β-actin and GAPDH.

To verify cellular production of miR-365, RNA was extracted from intact cells and screened using RT-PCR (Figure 1). Screening data confirmed the production of structural (beta actin) and metabolic (GAPDH) mRNA in all cell lines, as well as the presence of miR-365 among the oral cancer cells (SCC9, SCC15, SCC25, and CAL27) but not the normal OKF4 cell line. The levels of miR-365 appear to be much greater than the standardized control mRNAs for high-level transcripts needed for basic cellular functions, which may suggest the excess produced may be sufficient for export in extracellular vesicles or exosomes.

**Figure 2:**
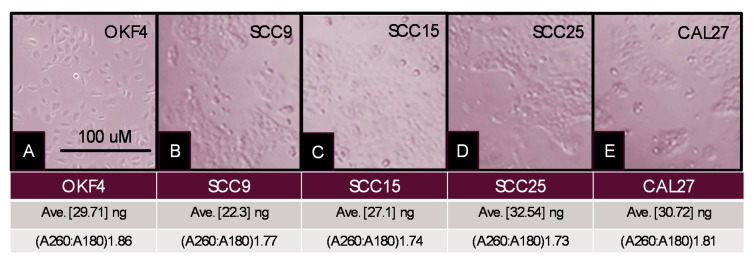
Cell culture and exosome isolation. (**A**) OKF4; (**B**) SCC9; (**C**) SCC15; (**D**) SCC25; and (**E**) CAL27 cell lines were established and subsequently cultured in exosome-depleted media for harvesting. Exosomes and extracellular vesicles were isolated from harvested supernatant revealing consistent RNA concentrations (range: 29.71–32.54 ng) and RNA purity (A260:A280 range: 1.73–1.86).

To evaluate exosomal content, the oral cancer cell lines (SCC9, SCC15, SCC25, and CAL27) and normal oral keratinocyte (OKF4) were grown in serum-free media, supplemented with exosome-depleted fetal bovine serum (Figure 2). Following exosome harvesting and separation, RNA was successfully isolated from the exosome-depleted supernatant from each cell line, averaging 29.71–32.54 ng. Relative concentrations of RNA were similar among each cell line (SCC9 average 29.71 ng; SCC15 average 27.1 ng; SCC25, average 32.54 ng; CAL27, average 30.72 ng; and OKF4 average 29.71 ng), which were not significantly different, *p* = 0.233. RNA quality was established by A260:A280 absorbance using a NanoDrop, revealing purity ranging 1.73–1.86.

**Figure 3:**
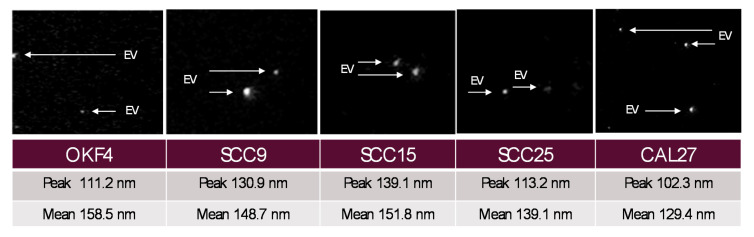
Exosome analysis using ZetaView Particle Metrix. The compositional analysis of exosome isolations demonstrated sizes ranging 50–300 nm, with an overall mean diameter of 134.8 nm and a peak analysis diameter of 124.6 nm, corresponding to known size distributions for extracellular vesicles (EV) and exosomes.

Confirmation of exosome and extracellular vesicle isolation was performed using the Particle Metrix Nanoparticle Tracking Analysis (NTA) and the manufacturer setting for extracellular vesicles “EVs” and nanospheres (Figure 3). In brief, isolated exosomes were diluted to achieve an Average Counted Particles per Frame (ACPF) below 100, well within the recommended particle per frame value range of 40–200. Measurements were made in successive cycles to verify the presence of extracellular vesicles (EVs) and nanoparticles ranging 50–300 nm in size. The peak EV analysis (largest number of particles of any particular size) was 124.6 nm, with an overall average EV mean diameter of 134.8 nm, which corresponds with known size distributions and parameters for exosomes and extracellular vesicles.

**Figure 4:**
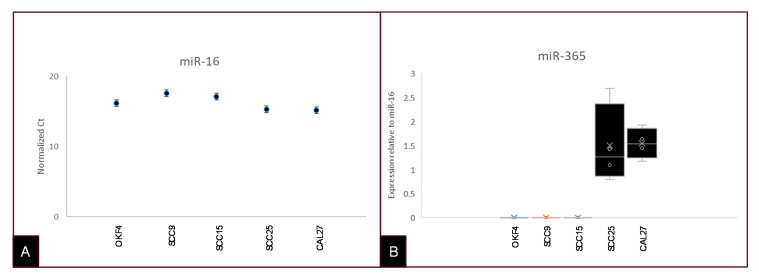
TaqMan microRNA assays for exosomal and extracellular vesicle-derived RNA. (**A**) Exosomal and extracellular vesicle RNA extracted from each cell line was screened using TaqMan assays for miR-16 (positive control) and miR-365. (**B**) Analysis of these data normalized to miR-16 expression revealed increased miR-365 relative to miR-16 and no expression of miR-365 among OKF4 or other cells.

Expression of miR-16 was subsequently used to confirm the presence of microRNA from the extracted exosomes and extracellular vesicles using qRT-PCR (Figure 4). Expression of miR-16 was normalized to RNA input (Figure 4A). The presence of miR-365 was then confirmed and normalized to miR-16 expression, which demonstrated an increased level of miR-365 in both CAL27 and SCC25 cells (Figure 4B). In addition, the normalized relative quantity (RQ) for miR-365 exhibited greater variation among SCC25 (1.382–4.363) than CAL27 cells (1.248–1.536).

**Figure 5:**
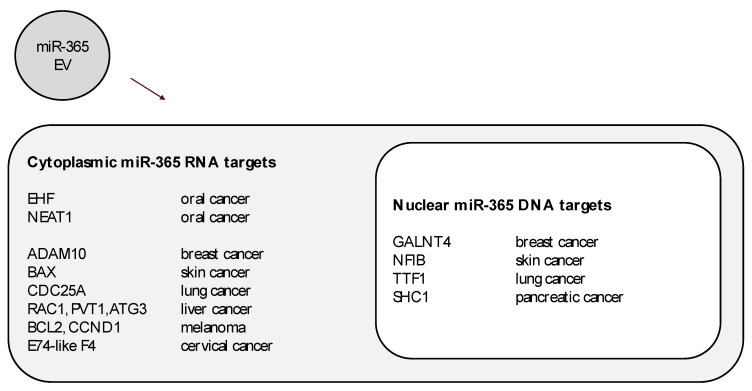
Cytoplasmic and nuclear targets of extracellular vesicle-derived miR-365. Some molecules known to be modulated by miR-365 expression in oral cancers may not include the full range of mRNA or DNA targets, as evidenced by miR-365 studies in other cancers such as breast, skin, lung, liver, cervical, and pancreatic cancers.

The confirmation of miR-365 in oral cancer exosomes may suggest that this miRNA, as a potential biomarker for salivary diagnostics and other types of liquid biopsies, may be useful to further research into the molecular targets that might be actively modulated by exogenous miR-365 (Figure 5). For example, although EHF and NEAT1 are known cytoplasmic targets of miR-365 regulation in oral caners, the potential for miR-365 to modulate other targets has been verified in additional cancers, such as breast skin, lung, liver, and cervical tumors. In addition, nuclear targets identified in other tumor types may yet provide targets to analyze for the potential for miR-365 modulation in oral cancers—although these studies have yet to be undertaken.

## 3. Discussion

A growing body of evidence has suggested various means for establishing reliable and effective biomarkers for liquid biopsy screenings [24,25]. Due to the variability in standardized biomarkers, such as oral human papillomavirus (HPV) infection [26,27], focus has recently been placed on validation of exosomal and extracellular vesicle-derived microRNAs as potential biomarkers for liquid biopsy cancer screening, including those of oral cancers [28,29]. Based on this information, the primary objective of this study was to determine if oral cancers produce exosomes or extracellular vesicles containing miR-365, which might be useful as a potential screening tool, as was recently demonstrated in advanced breast cancers [30,31].

These results are novel as previous studies have only validated the production and function of miR-365 in cellular studies of cytoplasmic and intra-nuclear pathways among oral cancers, but not exosomal or extravesicular export [13,14,15]. This study provides evidence of miR-365 exported into extracellular vesicles of oral cancers—and, as more studies confirm the presence of exosomal miR-365, the potential utility of this microRNA as a screening tool for salivary or liquid biopsies of oral cancers may become evident [32,33,34]. As models for highly-specific microRNA liquid biopsy detection become more detailed and expansive, studies that provide novel candidates for inclusion (such as miR-365 for oral cancer) are urgently needed to advance this diagnostic method [35,36].

However, despite the significance of these findings, there are limitations to this study that should be outlined. Due to temporal and financial constraints, only a limited number of oral cancer cell lines were evaluated, which have limited the range of cell lines examined and therefore the practical utility of these findings. In addition, this study only evaluated commercially available oral cancer cell lines and did not include any pre-malignant or malignant primary tumor samples. Future studies including this type of analysis could greatly improve the inferential quality and evidence regarding this microRNA.

These results confirm that miR-365 is not only expressed in oral cancer cell lines, but is also subsequently exported into exosomes and extracellular vesicles derived from these cultures. These data may help to contextualize the potential for this microRNA to contribute to the phenotypes and behaviors of oral cancers that express this microRNA. Future research will begin to investigate these potential mechanisms and pathways and to determine if miR-365 may be useful as an oral cancer biomarker for salivary or liquid biopsies.

## 4. Materials and Methods

### 4.1. Cell Culture

Four commercially available oral squamous cell carcinomas cell lines were obtained from American Type Culture Collection (ATCC), CAL27 (CRL-2095), SCC25 (CRl-1628), SCC15 (CRL-1623), and SCC9 (CRL-1629). The normal oral keratinocyte OKF4 was obtained from Lifeline Cell Technologies. CAL27 and OKF4 cells were maintained in Dulbecco’s Modified Eagle’s Medium (DMEM) with the addition of 10% Fetal Bovine Serum (FBS) and 1% penicillin–streptomycin (P/S). SCC25, SCC15, and SCC9 cells were maintained in 1:1 DMEM: Ham’s F12 media with the addition of 10% FBS and 1% P/S. All cells were maintained in a biosafety level-2 (BSL-2) humidified tissue culture incubator with 5% CO_2_.

### 4.2. Cellular RNA Extraction 

To confirm the cellular production of miR-365, RNA was isolated from each cell line using the RNA isolation kit from Invitrogen (phenol-based), according to the recommended manufacturer protocol. This involved the lysis of cells using 1.0 mL of reagent, transfer to microcentrifuge tubes, and the addition 0.2 mL of chloroform prior to incubation for five minutes. Samples were centrifuged at 12,000× *g* at 4 °C for 15 min. The upper nucleic acid-containing upper phase was transferred with the addition of 0.5 mL of isopropanol for RNA precipitation. Samples were mixed and centrifuged at 12,000× *g* for an additional five minutes. The supernatant was aspirated and the RNA washed with 75% ethanol prior to resuspension in 100 µL of sterile distilled water.

### 4.3. RT-PCR Screening 

Screening of RNA using reverse transcription PCR was accomplished using the following primers, synthesized by SeqWright Fisher Scientific (Fair Lawn, NJ, USA):

Beta actin forward, 5′-GTGGGGTCCTGTGGTGTG-3′; 18 nt, 67% GC, Tm: 69 °C
Beta actin reverse, 5′-GAAGGGGACAGGCAGTGA-3′, 18 nt, 61% GC, Tm: 67 °C
Glyceraldehyde 3-phosphate dehydrogenase (GAPDH)
GAPDH forward, 5′-ATCTTCCAGGAGCGAGATCC-3′; 20 nt, 55% GC, Tm: 66 °C
GAPDH reverse, 5′-ACCACTGACACGTTGGCAGT-3′; 20 nt, 55% GC, Tm: 70 °C
miR-365 forward, 5′-ATAGGATCCTGAGGTCCCTTTCGTG-3′; 25 nt, 52% GC, Tm: 70 °C
miR-365 reverse, 5′-GCGAAGCTTAAAAACAGCGGAAGAGTTTGG-3′; 30 nt, 47% GC, Tm: 72 °C

In brief, RT-PCR screening was performed using total RNA and the ABgene Reverse-IT One-Step RT-PCR Kit (Epsom, Surrey, UK) and an Eppendor mastercycler gradient thermocycler (Hamburg, Germany). One microgram of RNA was used for each reaction. Reverse transcription was performed for 30 min at 47 °C, followed by denaturation. Thirty cycles of PCR were then performed, using the standard program involving denaturation, annealing at the appropriate primer temperature for each primer pair, and 5 min of extension at 72 °C. PCR reaction products were visualized using 4% agarose gels and ethidium-bromide using a Kodak Gel Logic 100 Imaging system and 1D Image Analysis Software from Eastman Kodak (Rochester, NY, USA). PCR band densitometry and relative mRNA expression levels were quantified using Adobe Photoshop—Image Analysis tools.

### 4.4. Exosome Depletion Protocol

Due to the presence of high levels of endogenous exosomes in FBS that may interfere with the identification of cell-derived exosomes, cells were passaged into the appropriate media (DMEM or DMEM:Ham’s F12) containing 10% Exosome-Depleted FBS from Gibco (Fisher Scientific, Catalog A2720801; Fair Lawn, NJ, USA) for a minimum of 24 h, which removes >90% of bovine-serum derived exosomes, confirmed by NanoSight exosome staining, qRT-PCR, SDS–PAGE, and Western blot analysis [37]. Cell media was then replenished using the media containing 10% exosome-depleted FBS and cultured overnight prior to exosome isolation according the manufacturer protocol.

### 4.5. Exosome and Extracellular Vesicle Isolation Protocol

In brief, cell culture media was harvested and centrifuged at 2000× *g* or relative centrifugal force (RCF) for 30 min to remove cells and cellular debris. The supernatant was transferred to a sterile container leaving the pellet containing cells and cellular debris. To the supernatant, 0.5 volumes of Total Exosome Isolation reagent from Invitrogen were added and mixed by pipette until homogenous. Samples were incubated at 4 °C overnight (12 h) and subsequently centrifuged at 10,000× *g* (RCF) for 60 min at 4 °C. The supernatant was then aspirated and discarded leaving the exosome-containing pellet. The pellet was resuspended in 1× phosphate-buffered saline (PBS) and stored at 4 °C.

### 4.6. Exosome and Extracellular Vesicle RNA Extraction

In brief, the protocol for Total Exosome Isolation kit was accomplished following the manufacturer protocol, which included adding 1× PBS to each sample to bring the total volume to 200 µL. To each sample, an equal volume of 2× Denaturing Solution (200 µL) was added and mixed by vortexing and then incubated on ice for 5 min. Then, 400 µL of Acid-Phenol:Chlorofom were added and vortexed for 30–60 s and then centrifuged at 10× *g* (RCF) for 5 min.

The upper aqueous phase was removed and transferred to sterile microcentrifuge tubes with 1.25 volumes of 100% ethanol. Collection tubes were prepared with manufacturer-supplied filter cartons from the Total Exosome Isolation kit and 700 µL of each sample were then transferred into the appropriate filter and centrifuged at 10× *g* (RCF) for 15 s. Wash solution 2/3 (500 µL) was added to the filter and centrifuged for an additional 15 s and then this step was repeated prior to a final centrifugation at 10× *g* for 15 s. The filter was then transferred to a sterile collection tube and 50 µL of pre-heated Rnase water were added to the filter and centrifuged at 10× *g* for 30 s and this step was then repeated. The samples were then placed on ice or stored at −20 °C.

### 4.7. Exosome Analysis

Confirmation of exosome and extracellular vesicle isolation was performed using the Particle Metrix Nanoparticle Tracking Analysis (NTA) Zeta View software (Inning am Ammersee, Germany) and the manufacturer setting specific for the analysis of extracellular vesicles (EVs) and nanospheres. In brief, samples were diluted in sterile 1× PBS an approximate concentration of 3.2 × 10^7^ particles/mL to achieve Average Counted Particles per Frame (ACPF) below 100, well within the recommended particle per frame value range of 40–200. Measurements were made in successive cycles scanning with a minimum of 10 positions and the following settings: Focus, Autofocus; Scattering Intensity, Detected Automatically; Cell temperature, 28 °C. Analysis was completed using ZetaView Software 8.05.10 using the following the default analysis parameters.

### 4.8. Quantification of Exosomal RNA

Extracted RNA was measured using a NanoDrop spectrophotometer from Fisher Scientific (Fair Lawn, NJ, USA). In brief, measurements of absorbance were made at A260 nm and A280 nm to approximate both RNA quantity and quality. Quantity was determined using A260 nm absorbance with the dilution factor and Beer–Lambert extinction coefficient. Purity was measured using the ratio of A260:A280 nm with a correction from the reading at A230 nm for contamination.

### 4.9. cDNA Synthesis

Samples were prepared using the TaqMan Advanced miRNA cDNA synthesis kit and the protocol recommended by the manufacturer. In brief, samples were thawed, gently mixed, and briefly centrifuged. Poly(A) Reaction mix was prepared containing 10× Poly(A) buffer, ATP, Poly(A) Enzyme, and RNase-free water. To each well of the reaction plate, 2 µL of sample and 3 µL of Poly(A) Reaction mix were added and mixed. Each plate was centrifuged to spin down the contents and eliminate air bubbles and then placed into a thermal cycler for Polyadenylation at 37 °C for 45 min, followed by a stop reaction at 65 °C for 10 min.

In a separate microcentrifuge tube, Ligation Reaction Mix was prepared containing 5× DNA ligase buffer, 50% PEG 8000, 25× ligation adaptor, RNA ligase, and RNase-free water according to the manufacturer protocol. To each well of the reaction plate, 10 µL of Ligation Reaction Mix were added and then vortexed to mix and centrifuged to spin down contents. The reaction plate was returned to the thermal cycler for Ligation at 16 °C for 60 min.

In a separate microcentrifuge tube, Reverse Transcription (RT) Reaction Mix was prepared containing 5× RT buffer, dNTP mix, 20× Universal RT primer, 10 RT Enzyme mix, and RNase-free water according to the manufacturer protocol. To each well of the reaction plate, 15 µL of the RT Reaction Mix were added. Each plate was centrifuged to spin down the contents and subsequently placed into a thermal cycler for reverse transcription at 42 °C for 15 min and stop reaction at 85 °C for five minutes.

### 4.10. Amplification

To improve detection of low-expressing miRNA targets, cDNA from the reverse transcription may be amplified using the manufacturer amplification reaction. In brief, miR-Amp Reaction Mix was prepared containing 2× miR-Amp Master Mix 20× miR-Amp Primer Mix, and RNase-free water according to the manufacturer protocol. To a new sample plate, 45 µL of miR-Amp Reaction mix were added with 5 µL of the RT reaction product. Each plate was briefly mixed and centrifuged and then placed into a thermal cycler using one cycle of enzyme activation at 95 °C for 5 min, 14 cycles of denaturing at 95 °C and annealing/extending at 60 °C for 30 s, followed by the stop reaction at 99 °C for 10 min.

### 4.11. Real-Time PCR

Briefly, TaqMan Advanced Master Mix was prepared according to the manufacturer protocol, which included 2× TaqMan Fast Advanced Master Mix, 20× TaqMan Advanced miRNA Assay, and RNase-free water. Then, 15 µL of RT-PCR Reaction Mix were added to each well of the reaction plate with 5 µL of 1:10 diluted cDNA template. Each plate was briefly mixed and centrifuged and subsequently placed into a thermal cycler for enzyme activation at 95 °C for 20 s, and 40 cycles of denaturation at 95 °C for 1 s, and annealing and extension at 60 °C for 20 s.
